# A new role for an old actor: plant small RNAs orchestrate the phytobiome

**DOI:** 10.1093/ismeco/ycaf060

**Published:** 2025-04-14

**Authors:** Seonghan Jang, Choong-Min Ryu

**Affiliations:** Molecular Phytobacteriology Laboratory, Infectious Disease Research Center, Korea Research Institute of Bioscience and Biotechnology (KRIBB), Daejeon, 34141, South Korea; Molecular Phytobacteriology Laboratory, Infectious Disease Research Center, Korea Research Institute of Bioscience and Biotechnology (KRIBB), Daejeon, 34141, South Korea; Department of Biosystems and Bioengineering, Korea Research Institute of Bioscience and Biotechnology (KRIBB) School, University of Science and Technology, Daejeon, 34141, South Korea

**Keywords:** small RNA, microRNA, cross-kingdom RNA interference, rhizosphere microbiome, plant-microbe interaction

Small RNAs (sRNAs), including microRNAs (miRNAs) and small interfering RNAs in eukaryotes, serve as key regulators of gene expression in plants primarily through RNA interference (RNAi) [1]. Beyond their role in development and stress responses, plant sRNAs influence microbial interactions. They act as defence molecules against viruses and eukaryotic pathogens by targeting virulence genes [2, 3]. In addition, plants engage in sRNA-mediated communication with symbiotic fungi, where sRNAs facilitate mutualistic relationships by modulating fungal symbiosis (Fig. 1A) [4]. While cross-kingdom sRNA communication between plants and eukaryotic microbes is well established, recent findings demonstrate that plants can also transfer sRNAs to prokaryotes including rhizobial symbionts (Fig. 1A), plant pathogenic bacteria, and members of the rhizosphere commensal microbiome [5–7]. This transfer is proposed to occur as free sRNAs or via extracellular vesicles (EVs), although solid evidence confirming the uptake and functional role of sRNAs in bacterial cells remains limited [8].

**Figure 1:**
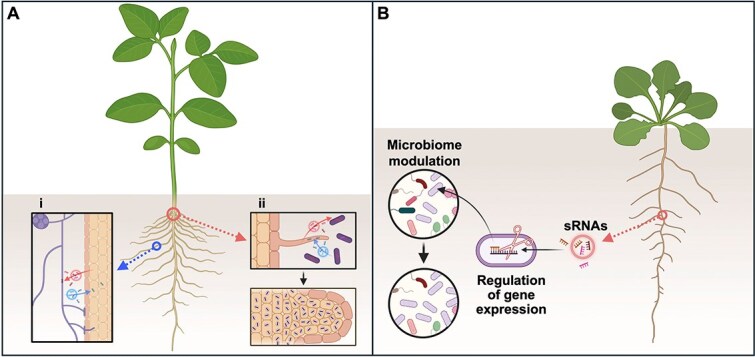
Cross-kingdom RNA communications between plants and microbes. (A) sRNA exchange in plant symbiotic relationships. (i) Mycorrhizal symbiosis. In arbuscular mycorrhizal interactions, the host plant and mycorrhizal fungi exchange sRNAs, modulating gene expression in both partners. This mutual regulation plays a crucial role in symbiosis establishment and nutrient exchange. (ii) Legume-rhizobia symbiosis. In legume plants, root-associated rhizobia exchange sRNAs with the host plant. This bidirectional sRNA communication regulates gene expression in both the bacteria and plant, promoting root nodule formation and nitrogen-fixing symbiosis. The efficient exchange of sRNAs enhances plant growth by optimising the symbiotic interaction. (B) Plant-derived sRNA-mediated microbiome modulation. Plants secrete specific sRNAs encapsulated in EVs or as naked forms into rhizosphere soil. These secreted sRNAs are taken up by rhizosphere-associated bacterial cells, where they regulate bacterial gene expression. The efficiency of sRNA-mediated gene silencing varies depending on the bacterial genus, ultimately leading to a shift in the rhizosphere microbiome composition towards a more beneficial state.

Recently, Middleton et al. (2024) provided the first clear evidence that plant-derived sRNAs are absorbed and utilised by rhizosphere bacteria [7]. This finding resolves previous uncertainties and demonstrates the role of miRNAs in regulation of bacterial gene expression and community structure (Fig. 1B) [7]. Once secreted, plant miRNAs are primarily absorbed by bacteria in a free state and modulate gene expression and selectively shape microbial composition, potentially contributing to plant health and growth. sRNA sequencing of rhizosphere soil detected distinct plant-derived miRNAs that are absent from bulk soil, indicating that plant miRNAs are actively secreted and do not passively accumulate [7]. In vitro treatment of bacteria with fluorescently labelled synthetic miRNAs further confirmed their uptake, demonstrating that plant miRNAs are effectively internalised in bacterial cytosol. However, bacterial transcriptional responses vary between species after miRNA uptake. While both *Variovorax paradoxus* and *Bacillus mycoides* are beneficial rhizosphere bacteria, their responses to plant-derived miRNAs differ. Only *V. paradoxus* exhibited notable transcriptional shifts, whereas *B. mycoides* showed minimal-to-no transcriptional changes, highlighting species-specific differences in miRNA processing. In *V. paradoxus*, miRNA exposure specifically modulated expression of genes associated with biofilm formation, antibiotic resistance, and motility, which are key traits essential for successful rhizosphere colonisation. These findings suggest that plant sRNAs selectively influence specific bacterial taxa, thereby contributing to the assembly and functional dynamics of the rhizosphere microbiome.

To further explore the role of plant miRNAs in microbiome regulation, Middleton and colleagues analysed rhizosphere microbiomes using *Arabidopsis* mutants with impaired miRNA biogenesis [7]. Such mutants exhibited reduced selective microbial filtering and increased microbial diversity, resulting in a microbial composition more similar to that of bulk soil. These results highlight that plant miRNAs contribute to microbiome assembly by shaping the bacterial community structure [7]. Furthermore, application of miRNA-encoded peptides, which enhance expression of specific miRNAs, to plants selectively altered the rhizosphere microbiome by modulating the abundances of key bacterial taxa.

To investigate the role of plant miRNAs in regulation of the rhizosphere microbiome under a controlled setting, Middleton and colleagues conducted an in vitro experiment using a simplified bacterial community comprising five agriculturally relevant taxa, *Acinetobacter*, *Enterobacter*, *Citrobacter*, *Pseudomonas*, and *Variovorax* [7]. Exposure to synthetic plant miRNAs induced a taxonomic shift, increasing the abundance of beneficial *Acinetobacter* while reducing the abundances of potentially detrimental *Enterobacter* and *Citrobacter*. This finding reinforces that plant miRNAs function as bioactive signals, reshuffling microbial composition by promoting beneficial microbes while inhibiting potentially deleterious bacterial taxa.

Despite these advancements, further research is required to gain a deeper understanding of the molecular mechanisms underlying cross-kingdom sRNA communication between plants and bacteria. While Middleton and colleagues clearly demonstrated that plant miRNAs influence bacterial transcription and communities [7], key questions remain unanswered regarding uptake, processing, and functional integration of sRNAs in bacterial cells. Answering these questions is crucial to comprehensively understand sRNA-mediated plant-bacteria communications and the broader complexity of sRNA exchange among diverse organisms in the phytosphere. The following questions outline key directions for further research.

***How are exogenous sRNAs processed in bacteria?*** The fate of these sRNAs in bacterial cells is unclear, although Middleton and colleagues clearly confirmed bacterial uptake of plant-derived miRNAs [7]. Unlike eukaryotes, bacteria lack a canonical RNAi machinery. Potential gene silencing mechanisms in bacterial cells include direct base-pairing with mRNAs, RNase-mediated degradation, and interactions with RNA chaperones like Hfq (Fig. 2A). Given the observed differences in the effects of sRNAs between species [7], different bacterial species likely possess distinct RNAi processing pathways or different translocation efficiencies, and this necessitates further investigation.

**Figure 2:**
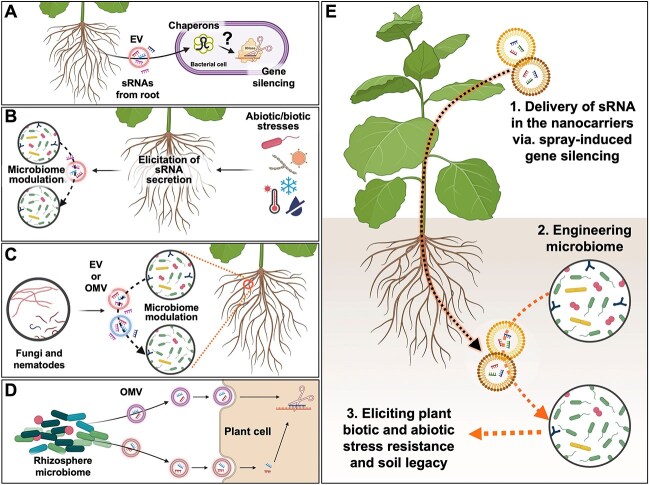
Potential mechanisms of plant-microbe sRNA communication. (A) Plant-derived sRNA uptake by rhizosphere bacteria. Plants release sRNAs into the rhizosphere encapsulated in EVs or in a naked state. After uptake by bacteria, these sRNAs may be stabilised by RNA chaperones (e.g. Hfq and ProQ) and regulate bacterial gene expression via RNase-mediated degradation. (B) Environmental regulation of plant sRNA secretion. Abiotic and biotic factors can stimulate production and secretion of plant sRNAs, shaping microbial communities for beneficial interactions. (C) sRNA exchange among soil microbes. Soil-dwelling eukaryotic and prokaryotic microbes can secrete sRNAs via EVs or OMVs, influencing the rhizosphere microbiome composition. (D) Bacterial sRNA transfer to plants. Rhizosphere bacteria may deliver their sRNAs to plants through OMVs. These sRNAs can enter plant cells via endocytosis of vesicles or direct membrane fusion, subsequently modulating plant gene expression. (E) sRNA application using nanocarrier-based delivery systems can enhance rhizosphere microbiome health by selectively suppressing harmful microbes and promoting beneficial ones, offering a sustainable strategy for plant growth and disease resistance. Additionally, these microbiome shifts can contribute to soil legacy effects, fostering long-term ecological balance and sustained benefits for plants across growing seasons.

***Do plants selectively secrete miRNAs to shape the phytobiome?*** Middleton et al. (2024) found that plants secrete specific miRNAs into the rhizosphere, which influences the microbiome. Under stress, plants may enhance selective sRNA secretion to recruit beneficial microbes while suppressing harmful taxa (Fig. 2B). Further research should determine whether plants dynamically adjust their sRNA profiles in response to environmental cues.

***Do rhizosphere bacterial sRNAs translocate into plants?*** Transfer of bacterial sRNAs to plants is possible. However, documented cases are scarce, and the underlying mechanisms are still unclear. Rhizosphere bacteria can release sRNAs via outer membrane vesicles (OMVs) or as naked sRNAs, which plants could internalise through endocytosis or an unknown mechanism (Fig. 2D) [9]. Future studies should examine bacterial sRNA stability in plant cells and determine whether uptake primarily occurs through OMV fusion or direct internalisation. Once delivered into plant cytosol, bacterial sRNAs could modulate plant physiology such as root exudation, potentially facilitating beneficial microbial colonisation.

***Can bacteria exchange sRNAs?*** Bacteria exchange sRNAs between the same and different species, influencing various interactions. The role of cytosolic sRNAs in bacteria is well known. Bacterial sRNAs can regulate and coordinate collective behaviours such as quorum sensing, stress responses, and virulence. Similarly, plant-associated bacteria may utilise sRNAs to enhance mutualistic interactions by promoting beneficial bacteria while inhibiting pathogens on the plant surface and inside plants. Identification of exchanged bacterial sRNAs and elucidation of their regulatory roles will be key to decipher microbiome dynamics.

***Can fungus- and nematode-derived sRNAs influence rhizobacteria?*** As members of the plant holobiont [10], pathogenic fungi and nematodes exhibit sRNA-mediated communication. Their eukaryotic sRNAs could also act as other signals that regulate bacterial gene expression, reshaping bacterial communities (Fig. 2C). Investigation of these interactions could reveal new dimensions of sRNA-based cross-kingdom microbiome regulation.

***What advantages does sRNA-mediated cross-kingdom communication offer over chemical signalling?*** Unlike broad-acting metabolites, sRNAs enable precise, target-specific gene regulation at minimal metabolic cost. sRNAs provide adaptive flexibility, allowing plants to fine-tune microbial interactions by directly modulating bacterial gene expression in a short amount of time. This targeted control may be more efficient than conventional chemical signalling, offering plants a means to dynamically shape the rhizosphere microbiome.

Expansion of sRNA research offers promising strategies for sustainable agriculture by enhancing plant defence and microbiome structuring. A recent study demonstrated that plant-induced bacterial gene silencing effectively utilises sRNAs as a biocontrol tool to regulate soil-borne bacterial virulence genes [6]. However, challenges remain regarding sRNA stability and delivery. Use of chemically synthesised nanocarriers such as lipid nanoparticles, liposomes, and artificial EVs could improve the longevity and targeting accuracy of sRNAs, allowing precise modulation of the rhizosphere microbiome (Fig. 2E). Moreover, sRNA-mediated microbiome structuring could contribute to long-term soil legacy effects, reinforcing plant health and resilience. Advancement of RNAi-based silencing, combined with nanotechnology, will be key to develop scalable, sRNA-driven agricultural solutions.

## References

[1] Weiberg A, Wang M, Bellinger M. et al. Small RNAs: a new paradigm in plant-microbe interactions. *Annu Rev Phytopathol* 2014;52:495–516. 10.1146/annurev-phyto-102313-045933 25090478

[2] Rössner C, Lotz D, Becker A. VIGS goes viral: how VIGS transforms our understanding of plant science. *Annu Rev Plant Biol* 2022;73:703–28. 10.1146/annurev-arplant-102820-020542 35138878

[3] Hamby R, Cai Q, Jin H. RNA communication between organisms inspires innovative eco-friendly strategies for disease control. *Nat Rev Mol Cell Biol* 2024;26:81–2. 10.1038/s41580-024-00807-y 39548286

[4] Ledford WC, Silvestri A, Fiorilli V. et al. A journey into the world of small RNAs in the arbuscular mycorrhizal symbiosis. *New Phytol* 2024;242:1534–44. 10.1111/nph.19394 37985403

[5] Middleton H, Dozois JA, Monard C. et al. Rhizospheric mirnas affect the plant microbiota. bioRxiv 2024:20220726501597. 10.1101/2022.07.26.501597 PMC11520407 39474459

[6] Jang S, Kim D, Lee S. et al. Plant-induced bacterial gene silencing: a novel control method for bacterial wilt disease. *Front Plant Sci* 2024;15:1411837. 10.3389/fpls.2024.1411837 39157516 PMC11327017

[7] Middleton H, Dozois JA, Monard C. et al. Rhizospheric miRNAs affect the plant microbiota. *ISME Commun* 2024;4:ycae120. 10.1093/ismeco/ycae120 39474459 PMC11520407

[8] Cai Q, He B, Wang S. et al. Message in a bubble: shuttling small RNAs and proteins between cells and interacting organisms using extracellular vesicles. *Annu Rev Plant Biol* 2021;72:497–524. 10.1146/annurev-arplant-081720-010616 34143650 PMC8369896

[9] Koeppen K, Hampton TH, Jarek M. et al. A novel mechanism of host-pathogen interaction through sRNA in bacterial outer membrane vesicles. *PLoS Pathog* 2016;12:e1005672. 10.1371/journal.ppat.1005672 27295279 PMC4905634

[10] Hassani MA, Durán P, Hacquard S. Microbial interactions within the plant holobiont. *Microbiome* 2018;6:1–17. 10.1186/s40168-018-0445-0 29587885 PMC5870681

